# Bad news itself or just the messenger? The high mortality of *Fusobacterium* spp. infections is related to disseminated malignancy and other comorbidities

**DOI:** 10.3402/ecrj.v3.30287

**Published:** 2016-05-10

**Authors:** Katrine Johannesen, Ram Dessau, Ole Heltberg, Uffe Bodtger

**Affiliations:** 1 Department of Respiratory and Internal Medicine, Naestved Sygehus, Næstved, Denmark; 2 Department of Clinical Microbiology, Slagelse Sygehus, Næstved, Denmark; 3 Institute for Regional Health Research, University of Southern Denmark, Odense, Denmark

**Keywords:** *Fusobacterium* infections, bacteria, anaerobic, bacteraemia, empyema, mortality

## Abstract

**Background:**

*Fusobacterium* species are pleomorphic, obligate anaerobic gram-negative bacilli. They are difficult to culture and grow slowly. If antibiotic treatment is initiated prior to blood cultures, the bacteria might evade detection. This is a comprehensive report on mortality in non-bacteraemia fusobacterial infection.

**Methods:**

Data were collected retrospectively in adults having a positive culture with *Fusobacterium* spp. admitted during 2000–2012 at the medical department. Data on culture specimens, number of cultures, admission and culture dates, patient age, gender, clinical disease, Charlson's index of co-morbidity, CRP level and survival were obtained. For comparison, we traced 60 consecutive, similarly obtained cultures from 2009 to 2010 containing *Staphylococcus aureus*.

**Results:**

Within a 12-year period, we identified 28 patients with a positive culture of *Fusobacterium* spp. in a medical ward serving a population of 220,000. Only a minority (39%) had a positive blood culture, and 54% had focus in respiratory tract or pleura. Overall 6-month mortality was 32%, and unrelated to subspecies, treatment or anatomic location but significantly related to age >60 years, admission for severe, acute illness, and comorbidity, especially metastatic malignancy. Comparison between infection with *Fusobacterium* spp. and *S. aureus* showed that *Fusobacterium* spp. infections were predominantly community acquired, while *S. aureus* were both community and hospital acquired. Overall mortality for both bacterial infections increased significantly with age and current malignant disease. *S. aureus–*infected patients carried a significantly higher mortality.

**Conclusion:**

Our data support that *Fusobacterium* spp. infection is a marker for significant, chronic disease rather than carrying a poor prognosis *per se*.

Summary at a glance: With this study we investigated *Fusobacterium* spp. infections and the mortality of these, as well as the comorbidities related to the mortality. We show that malignancy is related to a higher mortality in these infections specifically.

*Fusobacterium* spp. are pleomorphic, obligate anaerobic gram-negative bacilli that are found as part of the normal flora in human oropharynx, gastrointestinal tract and female genitalia. *Fusobacterium* spp. are difficult to culture, as they only grow under strict anaerobic conditions. They grow very slowly: visible growth on a solid medium is unlikely before 3–5 days (1). Standard treatment includes antibiotics – such as penicillin and metronidazole – as well as drainage (2–4). If cultures are made after initiation of routine, antibiotic therapy *Fusobacterium* spp. may thus evade detection.

*Fusobacterium* spp. have been associated with a diversity of diseases, mainly in the younger population: solitary lymph node abscess in toddlers, otitis media in children, peritonsillar abscess or bacterial tonsillitis in adolescents, sinusitis or tooth infection in children or adolescents, and dermal, subcutaneous or intraabdominal infections in adults (5). *Fusobacterium necrophorum* can, in rare cases, cause Lemierre's syndrome, a potentially life-threatening disease characterised by septic pulmonary embolism and vena cava thromboembolism (6–8). An overview of pathology of *Fusobacterium* spp. appeared recently (1). Why infections with *Fusobacterium* spp. turn invasive still remains unanswered, but theories suggest production of specialised bacterial toxins, or reduced host defence by viral or bacterial, pharyngeal co-infection (1, 9, 10). Despite an increase in *Fusobacterium* spp. bacteraemia being reported (2), it is still a rare finding: detected in less than 1% of >22,000 blood cultures (cumulated) (11–14), and in <10% of all pleural empyema cultures (15) in adults. Empyema *per se* carries a high mortality (16), and to the best of our knowledge, there are no comprehensive reports on mortality in non-bacteraemia fusobacterial infection.

## Methods

### Design

A retrospective study of all adult patients (>18 years old) having a positive culture with *Fusobacterium* spp., obtained during a hospital stay at our Department of Internal Medicine (Naestved Sygehus, Denmark) between 2000 and 2012. As comparator, we randomly selected a group with cultures positive for *Staphylococcus aureus*, obtained during a hospital stay at our Department of Internal Medicine (Naestved Sygehus, Denmark) between 2009 and 2010.

No patients were culture positive for both bacteria.

### Primary endpoint

Mortality at 30 days (1 month) after positive culture was obtained, stratified by bacteria and clinical data.

### Data

Our medical department serves a population of 220,000 inhabitants, including acute admission at all hours. During the study period, 14,645 subjects had ≥1 blood culture (*n*=16,145) or a pleural effusion culture (*n*=exactly 1,500). Data on culture specimens, number of cultures, admission and culture dates, patient age, gender, clinical disease, Charlson's index of co-morbidity and date of death were obtained from medical records. Malignancy was defined as a histopathological diagnosis of neoplastic disease (except non-melanoma skin cancer) – known at the time of culture, or diagnosed during the same hospital admission as the positive culture – and data were extracted from the National Database of Pathology. Data on mortality and malignancy were extracted by 15 December 2014. ‘Acute severe disease’ was defined as treatment in intensive care units, or any condition with a high risk for a fatal outcome within days, such as shock of any cause, large cerebral haemorrhages, acute coronary infarction, acute respiratory failure, acute renal or hepatic failure.

Two patients (blood culture positive for *F. necrophorum*) had missing clinical data (medical records destroyed) but demographic and microbiologic data and date of death were known.

### Cultures

For blood culture, three BacT/ALERT^®^ bottles (BioMérieux, Marcy l'Etoile, France) including one anaerobic bottle were used, according to the manufacturer, with 5.6 days of incubation and detection. *Fusobacterium* spp. were routinely identified by finding growth of gram-negative rods from the anaerobic sub-culture plates. If *Fusobacterium* spp. were suspected, cultures were examined for kanamycin susceptibility, green fluorescence in UV-light and smell of butyric acid. If these traits were present, further phenotypic methods were used for species identification according to a Danish guideline (17). However, only *F. necrophorum* or *F. nucleatum* were reported to the species level. Other isolates and isolates with ambiguous speciation were routinely reported as *Fusobacterium* spp., which was considered sufficient detail for the clinical routine. *S. aureus* was cultured according to the routine procedures and was identified simply from colony morphology, Gram stain and coagulase testing.

### Ethics

Data are presented anonymously. Due to study design, the study did not fall under the jurisdiction of the ethics committee system. However, the local ethics committee was notified. The study was registered with the Danish Data Protection Agency.

### Statistics

Statistical analyses were performed using commercially available software (SPSS version 20, IBM, USA), though survival analyses and regression analyses were performed using the survival package in the R software (www.r-project.org). Discrete data were presented as median (range), and binary data as percentage. Differences were examined with non-parametric testing (Mann-Whitney U-test resp. χ^2^-test). Missing data were omitted from analyses. Significance was reached when *p*<0.05. *P*-values >0.2 are denoted n.s. (not significant) in tables.

## Results

### *Fusobacterium* positive cultures

In total, 16,145 culture samples were analysed from our medical department, and merely 28 samples from 28 patients (male *n*=21, 75%; median age 65 [range 19–89] years) were positive for *Fusobacterium* spp.: *F. necrophorum* (blood cultures, *n*=4) including one patient with Lemierre's syndrome (described in (7)), and *F. nucleatum* (blood culture, *n*=1; pleural effusion, *n*=1). Culture sites were pleural effusions (*n*=7 out of 1,500; 0.5%), blood cultures (*n*=11 out of 16, 145; 0.07%), bronchial lavage at bronchoscopy (*n*=7), ascitic fluid (*n*=1), sputum (*n*=1) and groin abscess (*n*=1).

### Mortality

**Fig. 1:**
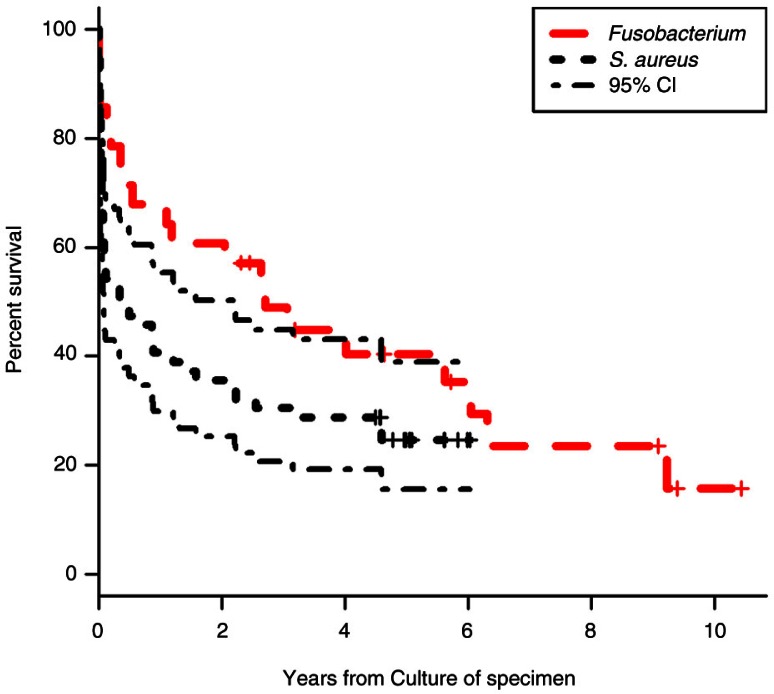
Survival in *Fusobacterium* spp. or *S. aureus* culture positive patients.

Mortality was significantly lower in patients’ culture-positive for *Fusobacterium* spp. (Table 1, Fig. 1), despite a higher prevalence of lung abscesses and pleural empyema, compared to the *S. aureus–*positive group (1 month: 14 *vs*. 37%; *p*<0.05; 6 months: 32 *vs*. 51%, *p*=0.1)

**Table 1:** Differences in demography, clinic, comorbidity and mortality stratified for focus of infection and type of bacteria

	Airway/lung/pleura, *n*=61			Blood/ascites/cavity, *n*=26			Overall airway *vs*. blood	Overall F (*n*=28) *vs*. S (*n*=59)
						
	Fusi, *n*=15	Staph, *n*=46	*p*	Fusi, *n*=13	Staph, *n*=13	*p*	*p*	*p*
Female, *n* (%)	2 (13%)	20 (44%)	0.06	5 (39%)	5 (39%)	n.s.	n.s.	0.12
Age, median (range)	65 (47–82)	69 (20–80)	n.s.	64 (19–89)	77 (41–89)	0.17	n.s.	0.09
Penicillin allergy, *n* (%)	4 (21%)	3 (6%)	0.033	5 (33%)	0 (0%)	0.16	0.12	0.005
Empyema/lung abscess, *n* (%)	9 (64%)	3 (7%)	0.0004	2 (17%)	0 (0%)	n.s.	n.s.	0.00005
Charlson ≤1, *n* (%)	6 (40%)	15 (33%)	n.s.	5 (39%)	4 (31%)	n.s.	n.s.	n.s.
Charlson, median (range)	2 (0–6)	2 (0–6)	n.s.	2 (0–6)	2 (0–7)	n.s.	n.s.	n.s.
Acute severe disease, *n* (%)	1 (7%)	25 (54%)	0.002	4 (33%)	7 (54%)	n.s.	n.s.	0.003
Active cancer, *n* (%)	6 (43%)	12 (26%)	n.s.	4 (33%)	4 (31%)	n.s.	n.s.	n.s.
Lung cancer, *n* (%)	5 (36%)	2 (4%)	0.006	0 (0%)	0 (0%)	n.s.	0.10	0.03
Mortality 1 month, *n* (%)	0	18 (39%)	0.003	4 (31%)	4 (31%)	n.s.	n.s.	0.03
Mortality 6 month, *n* (%)	3 (20%)	24 (52%)	0.038	6 (42%)	6 (42%)	n.s.	n.s.	0.10

Overall, *Fusobacterium* spp. was significantly less often associated with airway/pleura infections (54 *vs*. 78%; *p*<0.05), and even in patients with pleural empyema or lung abscess, mortality was significantly lower after 1 month compared to *S. aureus* (0 *vs*. 33%; *p*<0.05). No significant difference in 6-month mortality was observed. Likewise, 1- or 6-month mortality of non-empyema/non-lung abscess infection differed insignificantly between the fusobacteria-positive and the *S. aureus–*positive group (data not shown).

Overall prevalence of malignancy did not differ between groups (Table 1). However, lung cancer was significantly more prevalent in the fusobacteria-infected group (19 *vs*. 3%; *p*<0.05). Furthermore, the pattern of bacterial predominance and localisation of primary cancer differed significantly between patients with *Fusobacterium* spp. *vs. S. aureus* (Pearson χ^2^, *p*<0.05): head, throat or oesophageal cancers (4% *vs*. 10%), urogenital cancers (15% *vs*. 5%), colorectal cancers (4% *vs*. 3%) and other malignancy (breast, brain, lymphoma, pancreas: 0% *vs*. 9%).

Infection with *Fusobacterium* spp. predominantly appeared as a community-acquired infection compared to *S. aureus* infections with positive cultures obtained at median 0 (range 0–22) versus 3 (0–49) days after hospital admission (*p*<0.001).

Table 2 shows factors associated with mortality in univariate and multivariate analyses.

**Table 2:** Differences in demography, clinic, comorbidity and 6-month mortality in patients dead *vs*. alive 1 month after being culture-positive for fusobacteria or *Staphylococcus* aureus

	Dead within 1 month, *n*=61	Alive after 1 month *n*=26	*p*
Female, *n* (%)	22 (36%)	10 (39%)	n.s.
Age, median (range)	63 (19–86)	77 (51–89)	<0.0005
*Fusobacterium* spp., *n* (%)	15 (25%)	13 (50%)	0.02
Penicillin allergy, *n* (%)	7 (10%)	5 (16%)	n.s.
Empyema/lung abscess, *n* (%)	12 (21%)	2 (8%)	n.s.
Charlson ≤1, *n* (%)	5 (19%)	25 (41%)	0.051
Charlson, median (range)	2 (0–7)	3 (1–6)	0.003
Acute severe disease, *n* (%)	20 (80%)	17 (28%)	<0.0005
Active cancer, *n* (%)	18 (30%)	8 (32%)	n.s.
Lung cancer, *n* (%)	0 (0%)	7 (12%)	0.10
Airway/lung/pleura, *n* (%)	18 (69%)	43 (71%)	n.s.
Mortality 6 month, *n* (%)	100 (100%)	13 (21%)	<0.000001

Using regression analyses, increasing age and malignancy – but neither bacteria subtype (fusobacteria; *S. aureus*), pleura empyema/lung abscess, positive blood culture, gender, nor Charlson's index of comorbidity ≥2 – was significantly associated with mortality (data not shown).

In the fusobacteria subgroup, antibiotic treatment was administered to 22 patients; decided by discretion of the attending physician: intravenous penicillin and metronidazole (*n*=13, 46%), meropenem+ciprofloxacin;+metronidazole (*n*=2, both treated in Intensive Care Unit), cefuroxime+metronidazole (*n*=2, of whom one was allergic to penicillin) and penicillin monotherapy (*n*=5, recovering before culture result was available).

## Discussion

In our case series of adult patients with *Fusobacterium* spp. infections in a medical ward, we found a considerably higher 1- and 6-month mortality (14% *vs*. 32%) compared to previously reported overall mortality rates in Danish, medical wards (5% *vs*. 13%) with comparable age and comorbidity (18). However, our data suggest that host factors such as age and comorbidity, rather than fusobacterial infections *per se*, predicts mortality – both in airway/pleural infections and in septicaemia.

In 2012 Castellarin et al. reported an association between colorectal cancer and local *Fusobacterium nucleatum* gene-sequences present in tissue (19). In our study, colorectal cancer was a rare finding but our data suggest a clinically meaningful association between cancer anatomy and bacterial culture. *S. aureus* was more prevalent in cancers related with dysphagia, whereas *Fusobacterium* spp. was more prevalent in patients with disrupted mucosal barriers such as neoplasia in lung or urogenital tract (20, 21).

Few data exist on fusobacterial pleural empyema (15) but overall empyema mortality averages 20–40% (16). Four recent, retrospective series addressed mortality in patients with *Fusobacterium* spp. bacteraemia: 3-month mortality was 11% (13), 21% (14), 31% (12) and 41% (11). Despite differences in study populations (medical *vs*. surgical *vs*. ICU wards), all studies support our findings that Lemierre's syndrome is a rare manifestation of fusobacterial infection, and that *Fusobacterium* spp. infection mortality is primarily associated with age and comorbidity, rather than the anatomic location of *Fusobacterium* spp. infection, or treatment (4, 11–14). In our study, *Fusobacterium* spp. was associated with lower mortality at 1 and 6 months compared to a randomly selected population (size: >2×*n* fusobacteria-positive patients) culture positive for *S. aureus*, supporting that fusobacteria infections may be controlled in the acute setting, but should alert the medical team to possible, predisposing disorders. We chose *S. aureus* as comparator as it is a well-described, common pathogen (and everyday clinical challenge) found in the same compartments as fusobacteria (22). By choosing a single pathogen as comparator, we kept the head-to-head analyses simple; however, *Staphylococcus* has several immune evasion strategies which might confer the difference in early death (23) (Table 1).

A reliable *in vivo* assessment of treatment modalities would require a controlled study – hampered by the rarity of this disease entity. Only one other study has been looking at all *Fusobacterium* spp. regardless of anatomical location of infection: Pett et al. identified 18 patients in the United Kingdom between 1991 and 2013; however, 94% had bacteraemia, and the median age was lower. They found high mortality rates in the elderly and sick, and low (0%) in the young and/or *F. necrophorum* infections of the head/neck region (4).

We found a comparable, low prevalence rate of *Fusobacterium* spp.–positive blood cultures, but a much lower prevalence of *Fusobacterium* spp. pleural effusions (4). A policy in our department of microbiological examination of all pleural effusions – even those with a low *a priori* suspicion of infection – would tend to increase the denominator, decrease the prevalence. As fusobacteria are fastidious bacteria, culture facilities will determine successful detection rate. As in previous studies, we identified only a small *Fusibacterium* spp. positive patient cohort. Despite the low patient number, it seems unquestionable that age and underlying medical of conditions – acute or chronic – have a major impact, resulting in a decreased host defence and increased permeability of mucosa, precipitating the pathogenic potential of *Fusobacterium* spp., as an opportunistic pathogen (24).

The strength of the current study is the consistency and integrity of data: only one microbiological department and one clinical department with no loss of patients due to a unique person identification system. Despite the consistency of data, the major weakness of the study remains the small sample size. Detailed microbiological information on *Fusobacterium* spp.– except *F. necrophorum* or *nucleatum* – was not possible due to the retrospective design. A prospective study would provide more detailed data, but would be challenging due to the low prevalence of *Fusobacterium* spp. A controlled, clinical study of selected antibiotics may be considered, although arduous.

In conclusion, *Fusobacterium* spp. seems to be a messenger of clinically significant, acute or chronic illness, rather than implying pathogen-specific therapeutic challenges for the clinician.

In this retrospective and descriptive study, we found that infections with *Fusobacterium* spp. were rare, and mortality was related to comorbidity, primarily advanced or metastatic malignancy. Mortality was inferior to that observed in comparable patients infected with *S. aureus*.
